# The impact of the systemic inflammatory response on hepatic bacterial elimination in experimental abdominal sepsis

**DOI:** 10.1186/s40635-019-0266-x

**Published:** 2019-08-27

**Authors:** Katja Hanslin, Jan Sjölin, Paul Skorup, Frida Wilske, Robert Frithiof, Anders Larsson, Markus Castegren, Eva Tano, Miklos Lipcsey

**Affiliations:** 1 Anesthesiology and Intensive Care, Department of Surgical Sciences, Uppsala University, Uppsala, Sweden; 2 Section of Infectious Diseases, Department of Medical Sciences, Uppsala University, Uppsala, Sweden; 3 Hedenstierna Laboratory, CIRRUS, Anesthesiology and Intensive Care, Department of Surgical Sciences, Uppsala University, Uppsala, Sweden; 4 Section of Clinical Chemistry, Department of Medical Sciences, Uppsala University, Uppsala, Sweden; 5 Perioperative Medicine and Intensive Care, Karolinska University Hospital and CLINTEC, Karolinska Institute, Stockholm, Sweden; 6 Section of Clinical Bacteriology, Department of Medical Sciences, Uppsala University, Uppsala, Sweden

**Keywords:** Sepsis, Mononuclear phagocyte system, *Escherichia coli*, Endotoxins, Bacterial translocation, Animal models

## Abstract

**Background:**

Bacterial translocation from the gut has been suggested to induce a systemic inflammatory response syndrome (SIRS) and organ dysfunction. The liver has a pivotal role in eliminating circulating bacteria entering from the gut. We investigated whether pre-existing inflammation affects hepatic bacterial elimination.

**Methods:**

Fifteen anaesthetised piglets were infused with *E. coli* in the portal vein for 3 h. The naive group (*n* = 6) received the bacterial infusion without endotoxin exposure. SIRS (SIRS group, *n* = 6) was induced by endotoxin infusion 24 h before the bacterial infusion. For effects of anaesthesia, controls (*n* = 3) received saline instead of endotoxin for 24 h. Bacterial counts and endotoxin levels in the portal and hepatic veins were analysed during bacterial infusion.

**Results:**

The bacterial killing rate was higher in the naive group compared with the SIRS group (*p* = 0.001). The ratio of hepatic to portal venous bacterial counts, i.e. the median bacterial influx from the splanchnic circulation, was 0.06 (IQR 0.01–0.11) in the naive group and 0.71 (0.03–1.77) in the SIRS group at 3 h, and a magnitude lower in the naive group during bacteraemia (*p* = 0.03). Similar results were seen for hepatic endotoxin elimination. Peak log tumour necrosis factor alpha was higher in the naive 4.84 (4.77–4.89) vs. the SIRS group 3.27 (3.26–3.32) mg/L (*p* < 0.001).

**Conclusions:**

Our results suggest that hepatic bacterial and endotoxin elimination is impaired in pigs with pre-existing SIRS while the inflammatory response to bacterial infusion is diminished. If similar mechanisms operate in human critical illness, the hepatic elimination of bacteria from the gut could be impaired by SIRS.

**Electronic supplementary material:**

The online version of this article (10.1186/s40635-019-0266-x) contains supplementary material, which is available to authorized users.

## Background

The liver is an essential organ involved in the elimination of bacteria and bacterial products from the circulation and houses a substantial part of the mononuclear phagocyte system (MPS) implicated in this process. The human gastrointestinal tract houses several trillion microbial cells [1] crucial to normal functioning of the body but is also capable of causing severe infections. The liver is exposed to intestinal microorganisms and microbial fragments termed pathogen-associated molecular patterns (PAMPs) via the portal vein. Under healthy conditions, the liver acts as a gatekeeper preventing inflammatory triggers (e.g. bacteria and endotoxin) from entering the systemic circulation [2]. Kupffer cells, the resident macrophages in the liver, efficiently phagocytise pathogens and PAMPs entering the liver through the arterial or portal circulation or through both [3].

Bacterial translocation, defined as migration of viable bacteria or bacterial products from the gut lumen to normally sterile tissues [4], has been suggested to induce and maintain the systemic inflammatory response syndrome (SIRS) and multiple organ dysfunction syndrome (MODS) [5, 6], as well as serve as a source for bacterial infections [7]. While it is a matter of debate in sepsis and trauma, bacterial translocation has been described in liver cirrhosis [8] and in patients with intestinal obstruction [9]. In surgical patients, bacterial translocation is associated with systemic infectious complications [7, 10–12] and loss of gut barrier function may contribute to the development of MODS [13, 14]. Whether PAMPs entering the portal vein reach the systemic circulation depends on the barrier function of the liver, and although translocation of bacteria and PAMPs in severe illness has been extensively investigated, data on the barrier function of the liver are scarce.

The evidence on the development of immunosuppression and decreased bacterial clearance in sepsis is convincing [15–19]. We hypothesised that immunosuppression due to SIRS might also lead to depressed function in the hepatic MPS and thus to increased bacterial influx to the systemic circulation and subsequent escalated inflammation, assuming that bacterial translocation from the gut occurs in systemic inflammation.

The primary aim of the study was to investigate bacterial and endotoxin influx from the gut during bacteraemia to the systemic circulation in healthy piglets compared with piglets with pre-existing SIRS. Our primary endpoint was to study bacterial elimination by the liver during an infusion of live *Escherichia coli* (*E. coli*), measured as the ratio of hepatic to portal vein bacterial counts. Secondary aims were to investigate the hepatic elimination of endotoxin and the inflammatory response elicited by the *E. coli* infusion.

## Materials and methods

### Ethics statements

The experiment was approved by the Animal Ethics Board in Uppsala, Sweden (Dnr. C150/14). The piglets were handled in accordance with the Guide for the Care and Use of Laboratory Animals (EU Directive 2010/63/EU). ARRIVE and MQTiPSS guidelines were followed when relevant for these experiments [20, 21]. Some ARRIVE recommendations have been described by us previously and are not reported here [22]. MQTiPSS recommendations on replication and comparison of experiments in other animal with regard to species, comorbidities or sex were not performed for practical reasons. Neither was organ failure score or antimicrobial therapy used due to the nature and design of the experiments.

### Protocol

**Fig. 1:**
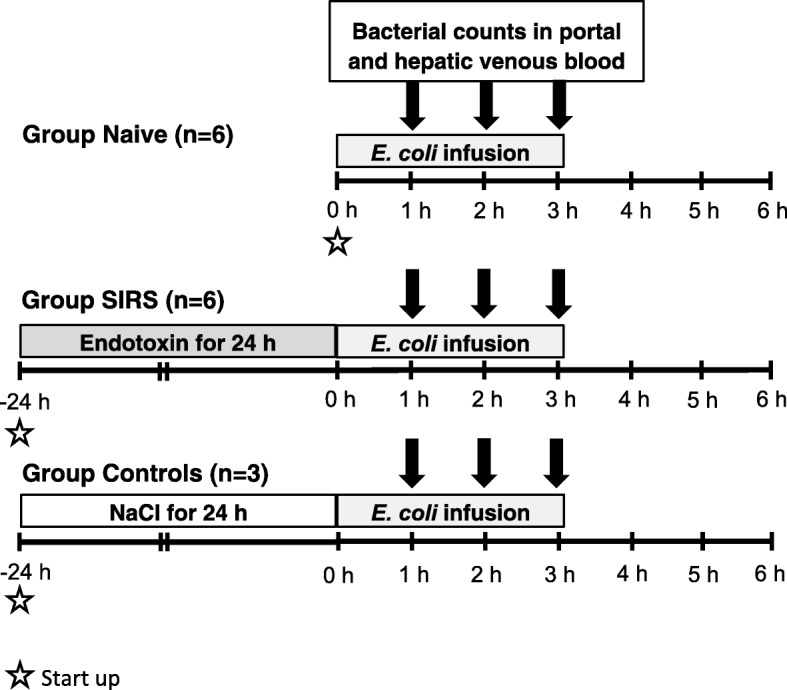
Overview of the study design. All animals were subjected to an infusion of *E. coli* in the proximal portal vein. The naive group received only the *E. coli* infusion. Animals in the SIRS group were given a continuous endotoxin infusion for 24 h before the *E. coli* infusion. Three piglets served as controls for 24 h anaesthesia and intensive care and received saline (instead of endotoxin) for 24 h before the *E. coli* infusion. Blood samples for the analysis of bacterial counts in portal venous, hepatic venous and arterial blood were collected hourly during the *E. coli* infusion

Fifteen Norwegian landrace breed piglets of both sexes, 8–10 weeks old, were anaesthetised and then catheterised for monitoring as described in Additional file 1: Supplement file. The animals were randomly assigned in blocks by blinded allocation to three experimental groups: naive (*n* = 6), SIRS (*n* = 6) and controls (*n* = 3). The study design is depicted in Fig. 1.

The *E. coli* (B09-11822 serotype O-rough:K1:H7; Statens Seruminstitut, Copenhagen, Denmark) used is a clinical isolate that is serum resistant and encapsulated. Fresh subcultures were prepared and grown into logarithmic growth phase before the experiment. To mimic bacterial influx from the gut *E. coli*, a common gut pathogen was infused into the portal vein. The microbiological methods were described previously [23]. In short, all animals were exposed to a continuous infusion of live *E. coli* (5 × 10^8^ colony-forming units [CFUs] in 25 mL saline) in the portal vein through the proximal catheter tip for 3 h.

To induce a mild SIRS, the animals in the SIRS group were exposed to intravenous endotoxin (*Escherichia coli*: 0111:B4; Sigma Chemical, St. Louis, MO) at 0.063 μg × kg^−1^ × h^−1^ [24] for 24 h before the *E. coli* infusion. The animals in the control group, who received saline instead of endotoxin before the start of the bacterial infusion, served as controls for the effects of 24 h of anaesthesia. All animals were followed for 3 h after completed *E. coli* infusion.

During the *E. coli* infusion, simultaneous blood samples for bacterial counts were taken hourly from the portal vein through the distal catheter tip, the hepatic vein and the artery. Bacterial concentrations were corrected for the weight of the animals and the infused *E. coli* dose. Bacterial counts during bacteraemia were determined by plating 0.1 mL blood in triplicate from the portal vein, hepatic vein and artery. *E. coli* was identified by colony morphology. To determine the pig blood bactericidal capacity, blood collected at 0 h was inoculated ex vivo with 10^5^ CFU × mL^−1^ *E. coli* in duplicate at 37 °C. Viable counts were plated hourly for 6 h. Bacteria-free endotoxin was analysed in plasma from the portal and hepatic vein at start-up in the SIRS and control group and at 0 and 3 h in all groups. Arterial blood was analysed regularly for blood gases, tumour necrosis factor-alpha (TNF-α), interleukin 6 (IL-6), interleukin 10 (IL-10) and complement. Immunosuppression was measured using the IL-10/TNF-α ratio [19, 25]. Complement activation was assessed with soluble TCC (sC5b-9).

The animals were treated according to a protocol to maintain vital parameters within pre-set limits (Additional file 1: Table S1). In short, arterial pressure of oxygen (PaO_2_) was maintained at > 10 kPa, MAP at ≥ 60 mmHg and cardiac output (CO) at ≥ 2 L × min^−1^. At the end of the experiment, all animals were culled by i.v. exposure to potassium chloride.

### Measurements

From 0 h, mean arterial pressure (MAP), mean pulmonary arterial pressure (MPAP), central venous pressure (CVP) and heart rate (HR) were continuously monitored. CO was measured with a Swan-Ganz catheter. Airway pressure values and respiratory volumes were recorded from ventilator readings. All physiological data were registered at intervals predetermined in the experimental protocol. Creatinine clearance was calculated by the conventional formula [26].

Blood from a cervical artery, pulmonary artery and hepatic vein were analysed for pH, gas tensions (PaO_2_, PaCO_2_), oxygen saturation, lactate, base excess and haemoglobin on an ABL^TM^ 800 and a Hemoximeter ^TM^ OSM-3 (Radiometer, Brønhøj, Denmark). Full blood count was analysed on a CELLDYN Sapphire (Abbott Scandinavia, Kista, Sweden). Plasma endotoxin was analysed in heparinised plasma with the chromogenic limulus amebocyte lysate assay (Endochrome-K; Charles River Endosafe, Charleston, SC, USA), and the lower detection limit in plasma was < 0.05 EU × mL^−1^. Plasma TNF-α and IL-6 were measured with porcine-specific sandwich enzyme-linked immunosorbent assays (ELISA; DY690B [TNF-α], DY686 [IL-6] and DY693B [IL-10], R&D Systems, Minneapolis, MN, USA). The limit of detection (LOD) in EDTA plasma was < 60 pg/mL for both TNF-α and IL-6. The LOD for IL-10 was 25 pg/mL. The ELISAs had total coefficients of variation of approximately 6%. Creatinine in urine and plasma was measured with enzymatic creatinine reagents (8 L24, Abbott Laboratories, Abbott Park, IL, USA) on a BS380 instrument (Mindray, Shenzhen, China).

For sC5-9 measurement, anti-Human C5b-9 (Diatec 5010; Diatec AS, Oslo, Norway) was used to capture antibody in sandwich ELISA [27]. Samples and calibrators were added to the wells. Bound sC5-9 was detected by biotinylated anti-C6 monoclonal antibody (Quidel, San Diego, CA, USA) and Streptavidin-HRP. The LOD for sC5-9 was 300 AU/L.

### Measurement of hepatic function

In a pilot study with the same set-up, hepatic function was assessed in 12 piglets by measuring indocyanine green (ICG) disappearance rate (ICG-PDR) [28].

### Calculations and statistics

Due to the lack of previous data on the ratio of hepatic to portal venous bacterial counts, we did not perform sample size calculation. A predefined analysis of data was done after six animals in the naive and the SIRS groups, and three animals in the control group. The statistical analysis was planned before the experiments and performed accordingly. Data were tested for normality. Data with a log-normal distribution were log-transformed. All values are expressed as mean ± SD or median (IQR) as appropriate, unless otherwise stated. For normally distributed data, Student’s *t* test was used for intergroup comparisons. ANOVA III for repeated measurements was used to assess group differences, change over time or group and time interaction, and if group differences were found, Unequal N HSD test was used as a post hoc test to identify between which groups the differences were found. For non-normally distributed data, the Mann-Whitney *U* test was performed for intergroup comparisons. Spearman’s rank correlation was calculated to test the potential associations between variables. All analyses were done using Statistica™ software (version 13.2, StatSoft, Inc., Tulsa, OK, USA). A p value of < 0.05 was considered statistically significant. The comparison of the control group with the SIRS group was performed in addition to the original statistical plan.

## Results

Additional file 1: Table S2 summarises the piglets’ characteristics at start-up. The baseline values were similar in the three groups. All animals survived throughout the experiment.

In the SIRS group, TNF-α and IL-6 levels peaked 2 h after the start of the endotoxin infusion (as a sign of SIRS). After 24 h of endotoxemia, i.e. at 0 h just before the start of bacteraemia, cardiac index (CI) was lower and arterial lactate higher. MAP was similar in the naive and SIRS group (Table 1).

**Table 1:** Physiological variables, norepinephrine dose and blood count during the experiment

Variable and time (h)	Naive	SIRS	Controls
Mean arterial pressure (mmHg)			
− 24		71 (± 6)	76 (± 13)
− 22		84 (± 6)	75 (± 11)
− 18		74 (± 9)	80 (± 14)
0	78 (± 11)	74 (± 9)	85 (± 10)
1	77 (± 10)	78 (± 8)*	80 (± 5)
2	77 (± 13)	91 (± 6)*	87 (± 30)
3	69 (± 11)	92 (± 7)*	95 (± 24)
4	81 (± 11)	92 (± 8)	94 (± 19)
5	76 (± 9)	88 (± 7)	77 (± 6)
6	75 (± 9)	86 (± 7)	63 (± 9)
Norepinephrine dose (μg × kg^−1^ × min^−1^)			
− 24		0 (0–0)	0 (0–0.07)
− 22		0 (0–0)	0 (0–0.07)
− 18		0 (0–0)	0.06 (0–0.07)
0	0 (0–0)	0 (0–0)	0.06 (0–0.29)
1	0.06 (0–0.13)	0 (0–0)	0.26 (0.1–0.29)*
2	0.03 (0–0.13)	0 (0–0)	0.26 (0–0.58)
3	0.19 (0–0.28)	0 (0–0)*	0.26 (0–0.58)
4	0.13 (0.06–0.51)	0 (0–0)	0.26 (0–0.58)
5	0.13 (0.06–0.26)	0 (0–0)	0.26 (0–0.58)
6	0.13 (0.06–0.14)	0 (0–0)	0.26 (0–0.58)
Cardiac index (L × min^−1^ × m^−2^)			
− 24		2.7 (± 0.5)	3.2 (± 0.2)
− 22		2.9 (± 0.4)	2.6 (± 0.3)
− 18		3.0 (± 0.6)	2.6 (± 0.6)
0	2.9 (± 0.9)	3.8 (± 0.6)	3.0 (± 0.4)
1	2.2 (± 0.4)	4.3 (± 0.5)***	2.7 (± 0.4)
2	2.7 (± 1.2)	3.6 (± 0.7)***	3.5 (± 0.6)
3	2.0 (± 0.2)	3.6 (± 0.7)***	3.6 (± 1.0)
4	2.0 (± 0.5)	4.0 (± 0.7)	3.6 (± 0.7)
5	2.2 (± 0.5)	3.8 (± 0.8)	4.0 (± 0.7)
6	2.5 (± 0.2)	4.4 (± 0.7)	4.4 (± 0.9)
Arterial lactate (mmol × L^−1^)			
− 24		1.8 (± 0.26)	1.1 (± 0.38)
− 22		1.2 (± 0.21)	0.7 (± 0.19)
− 18		0.8 (± 0.13)	0.7 (± 0.16)
0	1.2 (± 0.37)	0.8 (± 0.16)	0.8 (± 0.11)
1	2.2 (± 0.82)	1.0 (± 0.10)***	1.4 (± 1.9)
2	2.2 (± 0.27)	1.2 (± 0.21)***	1.9 (± 0.56)
3	3.0 (± 0.69)	1.1 (± 0.29)***	2.4 (± 0.32)
4	2.6 (± 1.2)	1.1 (± 0.34)	1.8 (± 0.81)
5	2.0 (± 1.2)	1.0 (± 0.27)	1.5 (± 0.76)
6	1.4 (± 1.1)	0.9 (± 0.19)	1.3 (± 0.81)

Values are expressed as mean (± SD), except the norepinephrine dose that is expressed as median (IQR)

Difference vs. the naive group 1–6 h assessed with ANOVA III for repeated measurements except for norepinephrine dose assessed with the Mann-Whitney *U* test

*p < 0.05

***p < 0.001

### Hepatic bacterial elimination

**Fig. 2:**
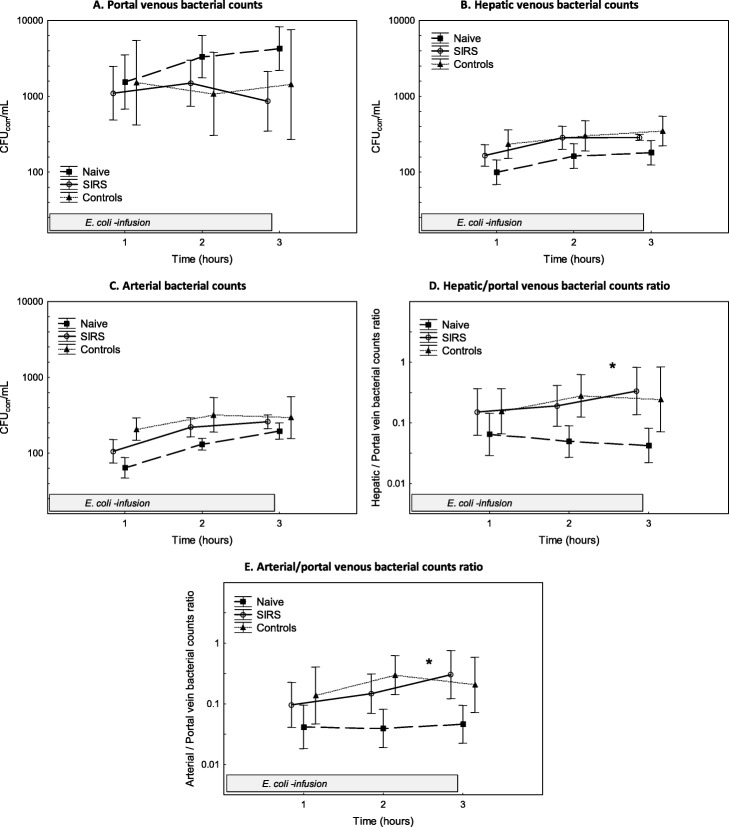
Bacterial counts in the distal portal vein, the hepatic vein and the artery during the *E. coli* infusion (**a**–**c**). The ratio of hepatic to portal venous bacterial counts and the ratio of arterial to portal venous bacterial counts during the *E. coli* infusion (**d**, **e**). Values are expressed as mean ± SEM (standard error of the mean). *p < 0.05. Difference between the SIRS and naive group as assessed with ANOVA III for repeated measurements

No bacteria were detected in arterial blood cultures taken at 0 h in the animals. The amounts of *E. coli* administered were comparable between the naive, the SIRS and the control groups (8.7 (± 0.2) vs. 8.8 (± 0.2) vs. 8.8 (± 0.2) log_10_ CFU). Figure 2 shows bacterial counts in the portal, hepatic venous and arterial blood during the bacterial infusion. The proportion of bacteria passing the liver circulation, i.e. the ratio of hepatic to portal venous bacterial counts, was lower in the naive group vs. the SIR group at 1–3 h (*p* = 0.03). In addition, the ratio of arterial to portal venous bacterial counts was lower in the naive group vs. the SIR group at 1–3 h (*p* = 0.049). There were no differences in bacterial counts between the control and naive group.

### Blood ex vivo bactericidal capacity

The blood bactericidal capacity ex vivo is missing for two animals in the naive group due to a technical error. The bactericidal capacity (Table 2) was higher in the naive animals, which is seen as lower bacterial counts in the naive (*n* = 4) vs. the SIRS group (*n* = 6; *p* = 0.001) and the naive (*n* = 4) vs. the control group (*n* = 3; *p* = 0.001). No bacteria could be detected in the samples of the naive group after 3 h, whereas bacterial growth was noted in the SIRS and the control group up to 6 h.

**Table 2:** Bacterial counts showing blood ex vivo bactericidal capacity

Time (h)	Naive group	SIRS group***	Control group***
0	5.0 (± 0.4)	4.9 (± 0.2)	4.9 (± 0.1)
1	1.1 (± 0.7)	3.0 (± 0.9)	3.5 (± 0.9)
2	0.0 (± 0.0)	2.4 (± 1.3)	3.4 (± 1.1)
3	0.3 (± 0.6)	2.0 (± 1.4)	3.2 (± 1.5)
4	0.0 (± 0.0)	1.7 (± 1.4)	2.7 (± 2.4)
5	0.0 (± 0.0)	1.3 (± 1.5)	2.8 (± 2.5)
6	0.0 (± 0.0)	1.2 (± 1.4)	2.9 (± 2.7)

Values are log-transformed and expressed as mean ± SD

Difference vs. the naive group 1–6 h assessed with ANOVA III for repeated measurements

***p < 0.001

### Hepatic endotoxin elimination

**Fig. 3:**
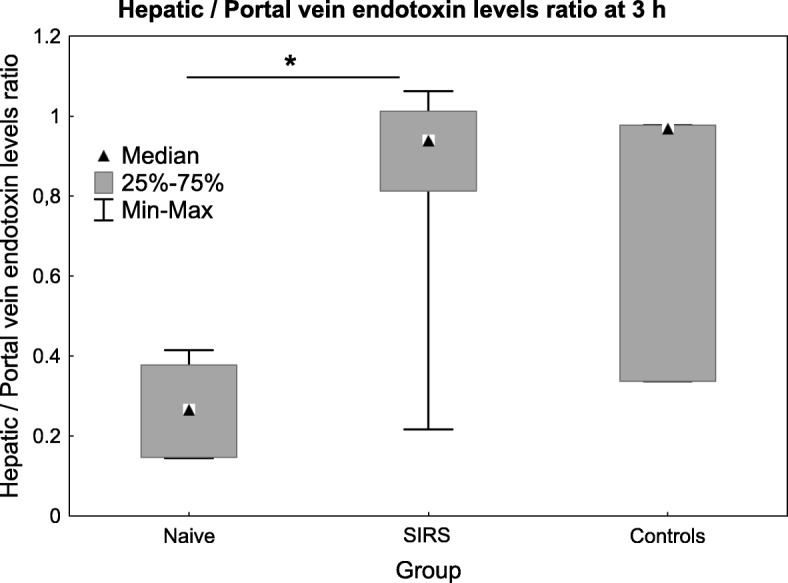
The hepatic to portal venous endotoxin ratio during the *E. coli* infusion at 3 h. Values are expressed as median (IQR). ***p < 0.001. Difference between the SIRS and naive group as assessed with the Mann-Whitney *U* test

Endotoxin levels were below detection limit in the portal vein and low in the hepatic vein at start-up in the naive (0.05 (0.05–0.05)) vs. SIRS group (0.05 (0.05–0.13) EU × mL^−1^). Endotoxin levels were below detection limit at both sites in the naive group at 0 h. In the SIRS group at 0 h, i.e. during ongoing endotoxin infusion, the endotoxin levels were below the detection limit in the portal vein and slightly elevated in the hepatic vein (0.11 (0.05–0.31) EU × mL^−1^). At 3 h, just before the end of the *E. coli* infusion, the endotoxin levels were higher in the naive group compared with the SIRS group in the portal (10.73 (8.2–15.3) vs. 1.64 (1.52–1.90), *p* = 0.02) and the hepatic vein (2.98 (2.69–3.06) vs. 1.70 (1.35–1.88) EU × mL^−1^, *p* = 0.005). Moreover, the ratio of hepatic to portal venous endotoxin levels, used as a measure of hepatic endotoxin elimination, was lower in the naive group compared with the SIRS group (Fig. 3, *p* = 0.03). There were no differences in endotoxin concentrations between the control and the naive group. The endotoxin levels in the portal vein at 3 h correlated with the portal venous bacterial counts (*ρ* = 0.73).

### Inflammatory, complement and circulatory response

**Fig. 4:**
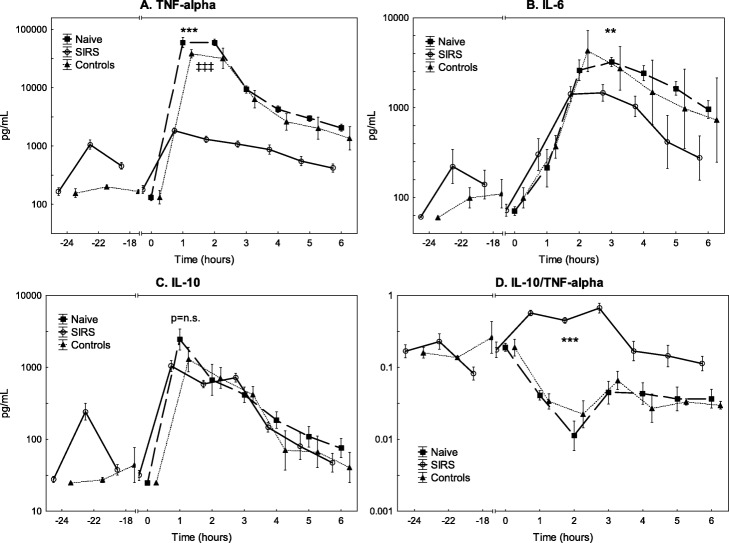
The levels of tumour necrosis factor alpha (TNF-α) (**a**), interleukin 6 (IL-6) (**b**), interleukin 10 (IL-10) (**c**) and interleukin 10/TNF-α ratio (**d**) during the experiment. All animals were subjected to an *E. coli* infusion for 3 h starting at 0 h. The SIRS group received endotoxin and the control group saline for 24 h before the bacterial infusion. Values are expressed as mean ± SEM (standard error of the mean). **p < 0.01, ***p < 0.001. Difference between the SIRS vs. naive group. ^‡‡‡^*p* < 0.001. Difference between the control vs. naive group. All assessed with ANOVA III for repeated measurements

The plasma TNF-α peaked 1 h after the start of the *E. coli* infusion while the IL-6 levels peaked 3 h after in both groups; the peak levels were higher in the naive group (*p* < 0.001) than in the SIRS group (*p* < 0.001) and the control group (*p* < 0.001; Fig. 4). Similar increases in IL-10 were observed in all groups during the experiment. The IL-10/TNF-α ratio increased markedly in the SIRS group but decreased in the naive group during *E. coli* infusion. This pattern remained throughout the experiment (*p* < 0.001).

**Fig. 5:**
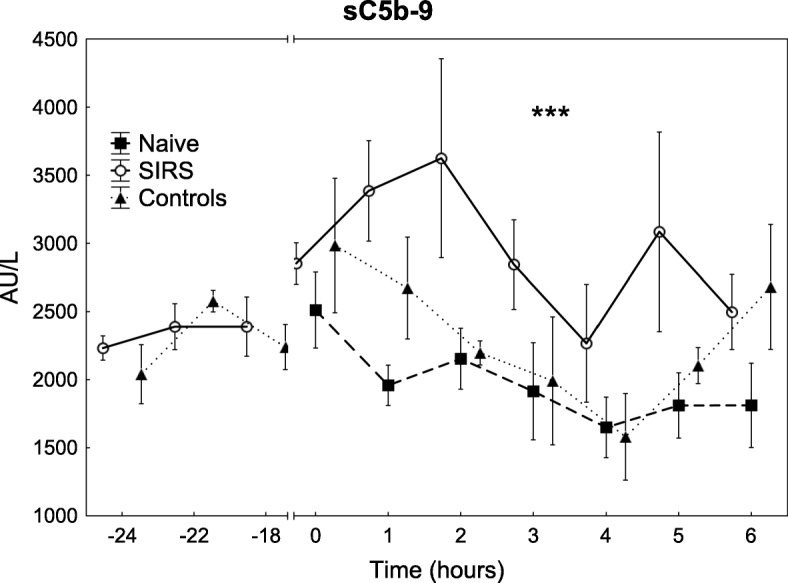
The levels of complement activation measured as sC5-9 during the experiment. All animals were subjected to an *E. coli* infusion for 3 h starting at 0 h. The SIRS group received endotoxin and the control group saline for 24 h before the bacterial infusion. Values are expressed as mean ± SEM (standard error of the mean). ***p < 0.001. Difference between the SIRS and naive group as assessed with ANOVA III for repeated measurements

Unlike the naive and the control groups, the SIRS group increased in sC5-9 levels during the *E. coli* infusion and had higher levels than the naive group during the experiment (Fig. 5, *p* < 0.001).

MAP was lower in the naive group compared with the SIRS group during bacterial infusion despite higher doses of norepinephrine (*p* = 0.01, Table 1). CI was lower and arterial lactate levels were higher during the *E. coli* infusion in the naive group compared with the SIRS group (*p* < 0.001 for both). No difference was seen in IL-6, IL-10, IL-10/TNF-α ratio, MAP, arterial lactate and sC5-9 levels between the control and naive group. TNF-α was lower and the dose of noradrenalin were higher in the control group compared with the naive group during the experiment.

### Estimation of liver function with indocyanine green

There was no difference in ICG-PDR between the naive and SIRS group at start-up (18.6 (± 6.4) vs. 21.4 (± 5.0), percent per minute). ICG-PDR was lower in the naive vs. the SIRS group (18.6 (± 6.4) vs. 32.4 (± 8.7), percent per minute, *p* = 0.02) at 0 h. No differences were seen between the control and naive group.

## Discussion

### Key findings

Hepatic bacterial elimination is very efficient in healthy animals but markedly impaired by even mild systemic inflammation. Likewise, endotoxin elimination by the liver is decreased in pigs with an ongoing systemic inflammatory response. Moreover, the bacterial killing capacity of the blood was notably reduced by mild systemic inflammation. The inflammatory response to an *E. coli* infusion, measured as peak levels of TNF-α and IL-6, was attenuated and showed an anti-inflammatory predominance measured as IL-10/TNF-α ratio, in pigs with relatively mild pre-existing systemic inflammation.

### Previous studies

The effects of inflammation-induced immunosuppression on bacterial elimination have previously been studied but with ambiguities and conflicting results. Preconditioning with endotoxin has been associated with augmented bacterial elimination from the circulation [29–31] and even with increased survival [29, 31]. The increased bacterial clearance after previous endotoxin challenge seen in these studies [29–31] seems to contradict our results. However, the immunological response after endotoxin preconditioning is dependent on the magnitude of the initial endotoxin challenge [32]. Moreover, timing of the secondary insult after the endotoxin challenge is a key factor in outcome [33] given that endotoxin tolerance diminishes over time. The high IL-10/TNF-α ratio in the animals pre-exposed to endotoxin in our study suggests that these animals were still in an IL-10-dominant immunosuppressed state. This is in line with the finding that IL-10 has been shown to mediate decreased bacterial clearance [30]. A study on isolated rat livers also demonstrated increased bacterial elimination and increased destruction of phagocytised bacteria after induction of systemic inflammation [34]. On the other hand, reduced bacterial clearance from blood and increased growth of bacteria in organs, including the liver, were reported in rabbits pre-exposed to endotoxin [35]. This study used intravenous infusion of bacteria, similarly to our report, that induces a more acute immune activation and may result in decreased bacterial clearance. Thus, the characteristics of both the primary and secondary insult seemingly affect bacterial clearance. In the present study focusing on hepatic elimination, we observed decreased elimination of *E. coli* by the liver in mild systemic inflammation. Both the degree and the duration of systemic inflammation before bacteraemia may be of importance, as delayed bacterial elimination, associated with increased growth of viable bacteria in organs, was more pronounced the longer the duration of systemic inflammation [35]. Furthermore, the effects of systemic inflammation on bacterial elimination may vary between organs. Our study specifically investigated hepatic bacterial elimination by the liver during pre-existing systemic inflammation.

The mechanisms underlying decreased bacterial elimination by the liver in systemic inflammation seen in our study are unclear. Several mechanisms in the liver are affected by sepsis [36]. In mice, hepatic bacterial clearance decreased during severe bacteraemia and mild bacteraemia developed into severe bacteraemia with increased mortality in the setting of Kupffer cell ablation. Similarly to our findings, decreased Kupffer cell function has been associated with increased systemic endotoxemia [37]. These findings illustrate the importance of these resident macrophages for bacterial elimination and that decreased hepatic bacterial elimination can affect outcome in bacteraemia [38]. We assessed liver function by ICG clearance [28], finding no signs of decreased liver function in animals exposed to endotoxin compared with the previously healthy ones before the *E. coli* infusion was started. This finding suggests that decreased hepatic bacterial elimination during systemic inflammation is most likely not explained by liver failure.

Hepatic endotoxin elimination was also decreased during *E. coli* bacteraemia in pigs with pre-existing systemic inflammation, corresponding to previous findings in mice hepatocytes [39]. Conversely, increased endotoxin clearance was reported after induction of endotoxin tolerance in rats [40]. Because we investigated global hepatic endotoxin elimination, the mechanisms underlying our findings are unclear. We demonstrated that pig blood is bactericidal on its own and contributes to total bacterial clearance. This bactericidal capacity was decreased in pigs with pre-existing systemic inflammation compared to healthy animals. Rapid killing of *E. coli* in the circulation and subsequent release of endotoxin may account for the high endotoxin levels measured in portal venous blood in the healthy animals with a high bacterial killing rate.

The differences in bacterial killing rate in blood between the groups are not explained fully by our findings. sC5-9 levels increased in the SIRS group and decreased in the naive group and were higher in the former group during bacteraemia. Since sC5-9 is essential for bacterial killing in blood [41], it is therefore unlikely that complement activation explains the observed decrease in bacterial elimination both in vivo and ex vivo in the SIRS group.

The physiological and inflammatory response to the bacterial infusion was diminished in pigs with pre-existing systemic inflammation, as manifested by only subtle changes in arterial blood pressure, arterial lactate and TNF-α levels. In contrast, healthy animals developed hypotension requiring noradrenalin treatment according to the protocol, hyperlactatemia and increased TNF-α levels. Although removal of bacteria and endotoxin by the liver was impaired in animals with pre-existing systemic inflammation, possibly leading to an increased systemic PAMPs load, it did not elicit an augmented inflammatory response in these animals. In this study, we found a substantially reduced bacterial killing capacity in the blood after endotoxin exposure for 24 h that corresponds to our previous finding that the inflammatory response of leukocytes to endotoxin is reduced [33]. Similar mechanisms may prevail in the phagocytic cells and neutrophils of the liver [42]. The reduced ability of the liver to eliminate bacteria and endotoxin during ongoing systemic inflammation, leading to increased inflow of these into the systemic circulation combined with a diminished physiological, inflammatory and metabolic response, could imply that the capability of the body to isolate and respond to abdominal bacterial infections is impaired in this condition. This contention could be of clinical relevance seeing that the association between an inflammation-induced decrease in phagocytic cell function and the increased risk of organ failure has been described in trauma patients [43]. Decreased elimination of PAMPs by the liver and the lower bactericidal capacity of the blood during systemic inflammation could also be phenomena consistent with sepsis-induced immunosuppression.

### Strengths and limitations

To our knowledge, this is the first study to evaluate the effects of systemic inflammation on bacterial elimination by the liver in vivo, as well as the first to describe diminished hepatic endotoxin elimination during systemic inflammation in a large animal model. The juvenile pig is large enough to allow instrumentation and monitoring used in intensive care units, making our model more clinically relevant than small animal models. Because organ support in itself affects the inflammatory response [44, 45], using an intensive care model also increases the clinical relevance of our study. Additionally, the porcine liver has similar anatomical, physiological and immunological properties as the human liver [46], and the circulation of the pig has been suggested to be most similar to that of humans among non-primates [47, 48]. Finally, we had a control group to describe the effects of 24 h of anaesthesia. The control group was similar to the naive group in most aspects; however, lower TNF-α levels and blood bactericidal capacity as well as a more hyperdynamic circulatory response to the *E. coli* infusion were seen in this group. Thus, the inherent effects of 24 h of anaesthesia were limited.

The study has several limitations. We conclude that bacterial elimination by the liver is impaired by systemic inflammation, but because no liver biopsies for cultures were taken, the bacterial killing capacity of the liver was not assessed. Because the concentration of bacteria in our samples is dependent on blood flow in both the portal vein and the hepatic artery, changes in the splanchnic blood flow could, in theory, have affected our results. Blood flow to the liver was not measured because in the pilot phase of the study instrumentation of the portal vein and hepatic artery to attach flow probes led to transient hepatic circulation impairments that could have affected bacterial elimination. However, given the magnitude of change in hepatic bacterial and endotoxin elimination, it is highly unlikely that our results depend on changes in hepatic blood flow.

### Clinical implications

Our data suggest that hepatic bacterial and PAMP eliminations are very efficient under healthy conditions, but even mild systemic inflammation could lead to increased inflow of bacteria and PAMPs into the systemic circulation. Concurrently, the ongoing SIRS limits the inflammatory, physiological and metabolic response of the body to these microbial triggers making it particularly vulnerable to microbial invasion. Moreover, these results imply that the microbial filter function of the liver fails in systemic inflammation, and that microbial spread via the splanchnic blood flow that in health would be eliminated by the hepatic MPS could enter the systemic circulation in severe illness and sustain systemic inflammation. Future therapeutic approaches to prevent or treat decreased hepatic elimination of PAMPs could be a novel way to tackle immunoparalysis in sepsis.

### Conclusions

Hepatic bacterial and endotoxin elimination is impaired by a systemic inflammatory response, and the physiological and inflammatory responses to bacteraemia are diminished in pigs with ongoing systemic inflammation. If similar mechanisms operate in the human inflammatory response, the hepatic bacterial elimination is impaired by systemic inflammation, allowing enteric bacteria to escape into the systemic circulation. Future studies should explore the cellular mechanisms of deceased hepatic bacterial and endotoxin elimination.

## Additional file

Additional file 1: Supplement file. **Table S1.** Experimental protocol. **Table S2.** Animals’ weight and physiological variables at start up. (DOCX 24 kb)

## Data Availability

The datasets used and/or analysed during the current study are available from the corresponding author on reasonable request.

## Abbreviations

ARRIVE: ARRIVE guidelines
CFU: Colony forming units
CI: Cardiac index
CO: Cardiac output
*E. coli*: *Escherichia coli*
ICG: Indocyanine green
ICG-PDR: Indocyanine green plasma disappearance rate
IL-10: Interleukin 10
IL-6: Interleukin 6
MAP: Mean arterial pressure
MODS: Multiple organ dysfunction syndrome
MPS: Mononuclear phagocyte system
PAMP: Pathogen-associated molecular pattern
PaO_2_: Arterial pressure of oxygen
SIRS: Systemic inflammatory response syndrome
Soluble TCC (sC5b-9): Soluble terminal complement complex (sC5b-9)
TNF-α: Tumour necrosis alpha
