# Supplementary material for: The impact of the systemic inflammatory response on hepatic bacterial elimination in experimental abdominal sepsis
Source: Intensive Care Med Exp. 2019 Aug 27;7:52. doi: 10.1186/s40635-019-0266-x (PMC6712186; doi:10.1186/s40635-019-0266-x)
Supplement: Supplementary file 1 — Supplement file. Table S1. Experimental protocol. Table S2. Animals’ weight and physiological variables at start up. (DOCX 24 kb) [file 40635_2019_266_MOESM1_ESM.docx]

**Supplement file**

*Animals*

15 Norwegian landrace breed piglets of both sexes, 8-10 weeks old, were housed in a farm and moved to the laboratory prior to the experiments.

*Anesthesia and fluid administration*

General anaesthesia was induced by injecting a mixture of tilétamin-zolazepam 6 mg x kg^-1^ and xylazin 2.2 mg/kg intramuscularly. Anesthesia was maintained with sodium pentobarbital 8 mg x kg^-1^ x h^-1^ and morphine 0.26 mg/kg/h dissolved in 2.5 % glucose, and rocuronium bromide 2.5 mg x kg^-1^ x h^-1^, both given as continuous infusions. The dose of rocuronium bromide was adjusted if shivering was detected. Acetated Ringer’s solution was administered; initially as a bolus 20 mL/kg shortly after induction of anesthesia, and thereafter 2 mL x kg-1 x h-1. This resulted in a total basal fluid administration rate of 10 mL x kg^-1^ x h^-1^, flushing of lines, and resuscitation as described below, not included.

*Preparatory procedure*

All preparations were performed under aseptic conditions. During the surgical preparations, group SIR and Controls received 750 mg cefuroxime intravenously (i.v.). A bolus dose of morphine 20 mg and ketamine 100 mg was given before the airway was secured by tracheotomy. The animals were mechanically ventilated throughout the experiment with a Servo I (Maquet Critical Care, Stockholm, Sweden). Initial respiratory settings were: respiratory rate 25 x min^-1^, inspiratory-expiratory ratio 1:2, inspired oxygen fraction (FiO_2_) 0.3, positive end-expiratory pressure (PEEP) 5 cmH_2_O and tidal volume (V_T_) 9 mL x kg^-1^. V_T_ was adjusted before the start of the protocol to result in an arterial pressure of carbon dioxide (PaCO_2_) of 5.0-5.5 kPa. A cervical artery was catheterized for pressure monitoring and blood sampling. A central venous line and a Swan-Ganz catheter were inserted through the right internal jugular vein into the superior caval vein and into the pulmonary artery, respectively. A 4F double lumen central venous catheter was placed in the portal vein through the splenic vein via left subcostal incision, and a 7Fr Swan-Ganz catheter was inserted to a hepatic vein through the left external jugular vein. The location of the hepatic and portal vein catheter tip was controlled using fluoroscopy. Performing a minimal vesicotomy, a urinary catheter was inserted into the bladder. A heating pad (Operatherm 200W; KanMed, Bromma, Sweden) was set to 38°C to decrease heat losses, and turned off after six hours or if the animals core temperature reached 42°C. After completed preparations, the animals were placed on their side and allowed to stabilize for 30 min before baseline values were registered and baseline blood samples collected. To prevent and treat atelectasis, alveolar recruitment maneuver was performed and the animal’s body position was changed every six hours.

**Table S1. Experimental protocol.**

| **Parameter** | **Threshold value for intervention** | **Interventions** |
| --- | --- | --- |
|  |  |  |
| PaO_2_ | <10 kPa first time | Increase FiO_2_ to 0.6 |
| PaO_2_ | <10 kPa thereafter | 1. Increase FiO_2_ to the next level: 0.6🡪0.8 🡪1.0, and |
|  |  | 1. Increase PEEP to the next level: 5🡪8🡪10🡪14 cmH_2_O, and |
|  |  | 1. Lung recruitment maneuver^a^ |
| PaO_2_ | >30 kPa | Decrease FiO_2_ to the previous level: 1.0🡪0.8🡪0.6🡪0.3 |
| PaCO_2_ | >6.5 mmHg | Increase V_T_ with 10 %, to maximum 15 mL x kg^-1^ |
| PaCO_2_ | <4.5 mmHg | If respiratory rate is ≤25, decrease V_T_ with 10 %. If respiratory rate is >25, decrease it with 10 %. |
| MAP | MAP = MPAP (<90 min after baseline or start of *E.coli*-infusion) | Single dose of 40 µg norepinephrine i.v. |
| MAP and/or CO | MAP<60 mmHg (>90 min after baseline or start of *E.coli* infusion) and/or CO<2.0 L x min^-1^. | Start norepinephrine infusion (20 µg x mL^-1^) 5 mL x h^-1^. If ongoing norepinephrine infusion, increase rate one step: 5🡪10🡪20🡪40 mL x h^-1^ |
| MAP | MAP = MPAP (>90 min after baseline or start of *E.coli*-infusion) | 1. Single dose of 20 µg norepinephrine i.v., and 2. Start norepinephrine infusion (20 µg x mL^-1^) 5 mL x h^-1^. If ongoing norepinephrine infusion, increase rate one step: 5🡪10🡪20🡪40 mL x h^-1^, and 3. Fluid bolus with acetated Ringer’s solution 15 mL x kg^-1^ |
| MAP | MAP>100 mmHg | If ongoing norepinephrine infusion (20 µg x mL^-1^), decrease rate one step: 40🡪20🡪10🡪5🡪0 mL x h^-1^ |
| Blood Glucose | <4.0 mmol x L^-1^ | Give 20 mL of 30 % glucose-solution i.v. |

**Table S2. Animals’ weight and physiological variables at start up**

| **Variable** | **Naive** | **SIRS** | **Controls** |
| --- | --- | --- | --- |
| Weight (kg) | 26.0 (± 1.1) | 25.8 (± 1.9) | 27.2 (± 5.3) |
| Mean arterial pressure (mmHg) | 78 (± 11) | 71 (± 6) | 76 (± 13) |
| Mean pulmonary arterial pressure (mmHg) | 17 (± 2) | 15 (± 3) | 19 (± 3) |
| Cardiac index (L x min^-1^ x m^-2^) | 2.9 (± 0.9) | 2.7 (± 0.5) | 3.2 (± 0.2) |
| PaO_2_/FiO_2_ | 64 (± 6) | 63 (± 2) | 62 (± 3) |
| Static compliance | 32 (± 8) | 28 (± 7) | 40 (± 14) |
| Arterial lactate (mmol x L^-1^) | 1.2 (1.1-1.3) | 1.8 (1.4-1.9) | 1.1 (1.1-1.7) |
| Base excess (mmol x L^-1^) | 5.9 (± 1.2) | 5.4 (± 1.2) | 6.2 (± 1.7) |
| pH | 7.50 (± 0.03) | 7.49 (± 0.03) | 7.49 (± 0.02) |
| Leukocyte count x 10^9^ x L^-1^ | 13.8 (± 2.1) | 12.6 (± 2.8) | 13.9 (± 4.6) |
| Neutrophil granulocyte count x 10^9^ x L^-1^ | 6.5 (± 2.0) | 6.0 (± 2.7) | 7.8 (± 3.1) |

Values are expressed as mean ± SD, except arterial lactate, which is expressed as median (IQR)
